# Moe-Phobia: Effect of Users' Gender on Perceived Sexuality and Likability Toward Manga-Like Virtual Agents

**DOI:** 10.3389/fpsyg.2022.752748

**Published:** 2022-05-09

**Authors:** Tetsuya Matsui

**Affiliations:** Department of Robotics, Faculty of Robotics and Design, Osaka Institute of Technology, Osaka, Japan

**Keywords:** human-agent interaction, virtual agent, gender bias, moe-phobia, manga-like character

## Abstract

In Japan, many incidents regarding manga-like virtual agents have happened recently, in which critics have indicated that virtual agents used in public spaces are too sexual. Prior study defined this perception as “moe-phobia.” In many cases, critics have pointed to agents' clothes. However, after verifying actual moe-phobia incidents, I hypothesize that these incidents are associated with not only the agents' clothes but also the situations in which they are used. I conducted an experiment with three factors and two levels to verify this hypothesis. The independent values were the agents' clothes, usage scenario, and the gender of the participants. The dependent values were the agents' trustworthiness, familiarity, likability, sexuality, and suitability as perceived by humans. I conducted the experiment with female and male groups and conducted a three-way ANOVA for each dependent value for each group. As a result, I observed a different tendency regarding the impression of the agents between female and male groups; however, both groups had the same tendency regarding the perceived suitability. The female and male participants judged the agents' suitability from not only their clothes but also the scenario.

## 1. Introduction

In this article, I focused on the notion of “moe-phobia,” which is disgust toward female manga-like virtual agents who are perceived as sexual. “Moe-phobia” is one problem regarding the gender of agents and users. This word was suggested by Ito (Azuma, 2003). Ito defined it as the tendency to deny one's own feelings about being attracted to a virtual character. Later, Ito extended the notion of this word and included the intense disgust toward female virtual agents and the desire to ostracize these agents (Ito, 2008). In this article, I use “moe-phobia” in the latter meaning. This intense disgust toward virtual agents can cause systems that use virtual agents to be rejected, which is, thus, a large problem for HAI (human-agent interaction). The research question is how an agent's appearance and context affect moe-phobia.

“Moe” is originally a Japanese slang word meaning the strong positive emotions felt toward fictional characters, especially girl-like manga characters (Galbraith, 2009). Today, manga-like female characters appearing in fiction and media are generally called “moe-characters” in Japan. In this article, I use the term “moe-phobia” instead of simply “negative feelings toward female manga-like characters” for the following reasons. First, many public and private organizations in Japan have used moe-characters for public relations (PR) even though almost all controversies regarding female manga-like characters have been caused by moe-characters. These usages and controversies seem to have been caused by a divergence in people's impression of the concept of “moe.” Thus, I focus on this divergence, especially between genders. Second, Ito suggested the notion of “moe-phobia” in research on Japanese fiction culture (Ito, 2008). Limniati pointed out that Japanese manga and anime affected real human-robot interaction and human-agent interaction (Limniati, 2017). Also, the appearance of virtual YouTubers seems to have been influenced by Japanese manga and anime. Thus, the notion of “moe-phobia” could be transferred from research on manga and anime to HAI.

Manga-like virtual agents are widely used on the web and in the real world. On the web, virtual YouTubers are avatars that are agents operated by streamers and who act as real celebrities (Shirai, 2019; Zhou, 2020; Lu et al., 2021). The number of them is higher than 13,000 as of December 2020 (TEC, 2020), and together, they have earned more than 4,00,000 dollars in total *via* Super Chat (PLA, 2020). Many virtual YouTubers are human-like female virtual agents who look like Japanese manga characters.

Also, female virtual agents are widely used in the real world. “Mei” is a Japanese human-like female agent that was developed by the Nagoya Institute of Technology, and Mei appears on digital signage as a guide for the campus (Lee et al., 2013). “Hatsune Miku” is an animated character from an ordinary voice synthesis software package; however, the 3D model was constructed and is used to perform in concerts as a singer (Leavitt et al., 2016; THEIR, 2016), and Hatsune Miku is also used as a tourism promotion character in Sapporo Endo (2016). KDDI constructed “Rena,” a virtual character operated by character AI and executed on an XR system, and Rena was used to promoting Iida city (KDD, 2018). In these cases, virtual agents are used for regional vitalization. In Japan, many virtual characters are used for the same roles, and they are called “local moe-characters” (Kim, 2017). They are often produced by local authorities and local companies. Many of them are manga-like female virtual characters in Japan.

These agents and characters have often been accepted and embraced by the media and people and, thus, have a large effect on society. However, in some cases, they get flamed on the web, and there are various discussions with respect to gender bias and sexual expression (refer to Section 2).

In the research field of HAI, other practical uses of virtual agents have been widely researched, e.g., in the role of medical counselors (Parmar et al., 2018; Wang et al., 2021) and for product recommendation (Qiu and Benbasat, 2009; Matsui and Yamada, 2019). In these cases, female virtual agents are often used. Thus, investigating why some people are disgusted by female virtual agents is an important problem for HAI.

Flaming on the web is a serious problem on recent social networking sites (SNSs) (Moor et al., 2010; Helfrich, 2014; Jane, 2015; Hwang et al., 2016). This is a phenomenon in which an avalanche of negative comments or slander appears in response to particular news or people. Typically, flaming arises and spreads on SNSs and often leads to serious results, crime, and leaking of private information (Mori and Takeda, 2019). Moe-phobia often leads to flaming on the web (refer to Section 2); however, the fundamental reason for this has not been sufficiently researched.

Many pieces of prior study focused on agents' and participants' gender in HAI. However, these studies often had contradictory results. Payne et al. showed that female users prefer interacting with agents of the same gender, whereas male users choose an agent of different gender (Payne et al., 2013). Guadagno showed that users prefer agents of their own gender (Guadagno et al., 2007). Kim et al. showed that users (male and female) had an impression of male personified agents as being closer to humans than female personified agents (Kim et al., 2007). These results show inconsistency, demonstrating that the gender of agents and users is a difficult problem to approach with experiments.

In addition, it has been reported that what an agent and human wear affects the interaction. Küster et al. showed that humans judged virtual agents by what they wear without other non-verbal information (Küster et al., 2019). Their study used female and male virtual agents. Fox and Bailenson conducted an experiment with suggestively and conservatively dressed female virtual agents in virtual reality (VR) and found that gender-stereotypical virtual women enhance negative attitudes toward women (Fox and Bailenson, 2009). Wang and Yeh investigated the effect of a female pedagogical agent with sex appeal. They showed that this agent was effective at getting users to study but was perceived as being untrustworthy and unprofessional. These prior studies suggest that gender bias will affect HAI.

Sexual appearance and symbols have often been shown to have a positive effect on advertisements. Wirtz et al. conducted a meta-analysis on studies of sexual appeal in advertisements and found that this appeal had a positive effect on male customers (Wirtz et al., 2018). Huo and Yuan showed that sexual objects (e.g., a female model in swimwear) in advertisements increase the buying motivation of male customers (Huo and Yuan, 2017). These advertisements rarely cause flaming on the web like in the case of female virtual agents in Japan. This could indicate that flaming on the web is unique to these agents. On the other hand, the sexual appeal does not always bring about a positive effect. Daniels and Zurbriggen showed that girls and young women on Facebook that displayed sexualized profile photos were considered as being less physically attractive, less socially attractive, and less competent to complete tasks (Daniels and Zurbriggen, 2016).

In this article, I first discuss cases of flaming involving virtual agents and characters in Japan to verify what in particular many people have a problem with: the female virtual character itself, sexual expression, or the social context (aims and situations for which agents or characters are used).

I hope that this article can contribute to avoiding cases of flaming involving virtual agents and characters and suggest a design method for female virtual agents.

## 2. Actual Cases of Moe-Phobia

### 2.1. Kizuna AI Incident

**Figure 1:**
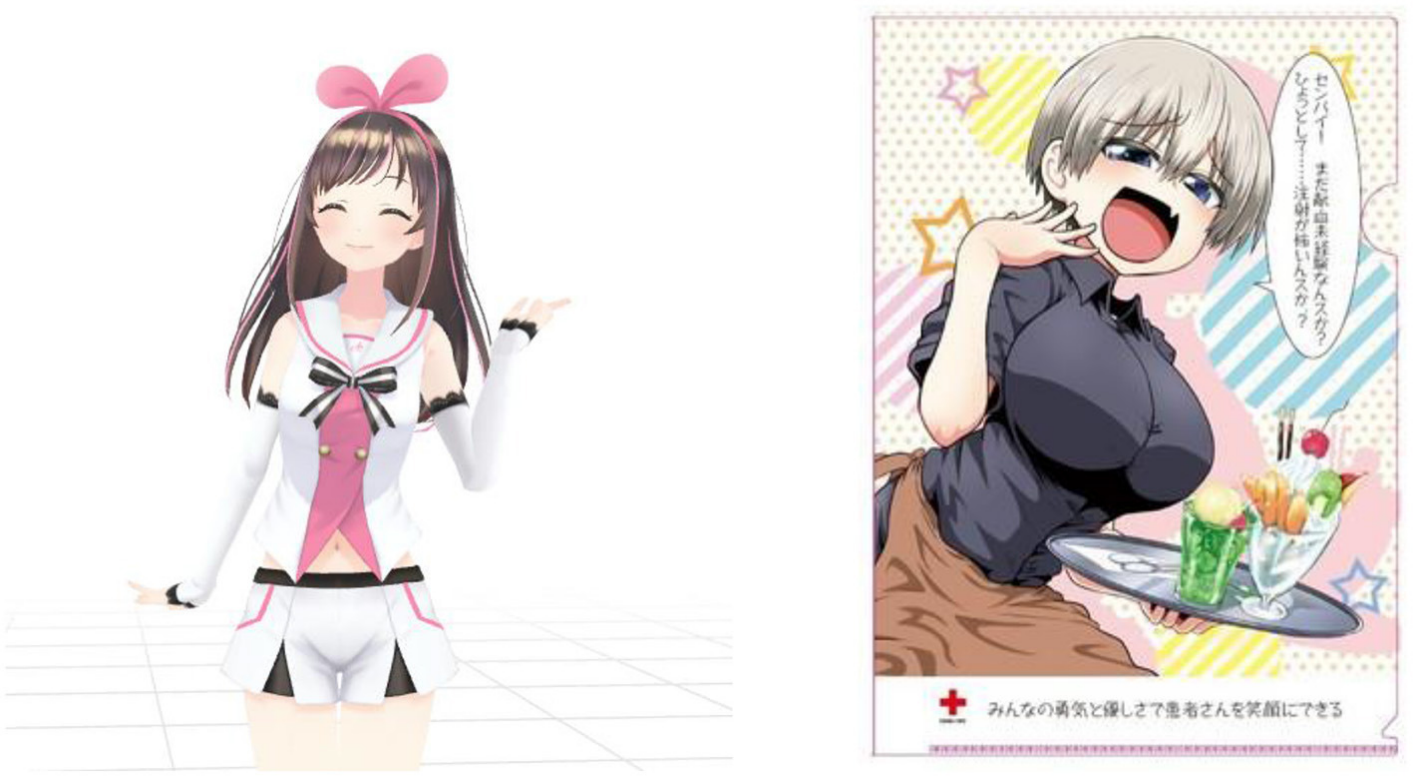
Left: Kizuna AI (https://kizunaai.com/). Right: Uzaki-chan's collaboration art with Japanese Red Cross for blood donation campaign. Adapted/Reproduced with permission from IVR, available at https://vkatsu.jp/.

Kizuna AI (left character in Figure 1) is a human-like female virtual YouTuber who mainly broadcasts gameplay and songs (Zhou, 2020). In October 2018, NHK (Nippon Hoso Kyokai, Japan Broadcasting Corporation) used Kizuna AI as a commentary character for news on the Nobel Prize. After this broadcast, some critics criticized this program on SNS (Ando, 2018; Senda, 2018; Korenaga, 2019). Roughly speaking, they had two complaints:

- Kizuna AI's design is overly sexual (for example, Kizuna AI wears short pants).
- In this program, Kizuna AI only listened to the commentary, which could perpetuate gender role divisions.

Critics pointed out that Kizuna AI was not suitable for NHK's TV program for these two reasons (Ando, 2018). The important point is that NHK is a public broadcasting station in Japan. Many critics said that a “public” organization should not use moe-characters like Kizuna AI (Ando, 2018).

### 2.2. Ekino Mochika Incident

“Ekino Mochika” is a PR character for Tokyo Metro (TOMYtec, 2016). Ekino Mochika is also a human-like female virtual character. Tokyo Metro has used this character since 2013, and in 2016, a new concept design was released in collaboration with TomyTec Co., Ltd. (Amio, 2016). Since this design was released, criticism has increased on the web (Amio, 2016). The main criticisms have focused on the appearance of her clothes design, especially on the leg line showing through her skirt. Critics have focused on two points: this design can evoke sexual content, and this character is not suitable for public transportation (Amio, 2016).

### 2.3. Uzaki-chan Incident

“Uzaki-chan Wants to Hang Out!” is a Japanese comic, and “Uzaki-chan” is the heroine of this comic. In October 2019, the Japanese Red Cross Society appointed her to a poster for promoting blood donations (right picture in Figure 1). As a result, some people criticized this poster because it looked overly sexual (Ariyoshi, 2019). On the poster, Uzaki-chan was completely clothed, but critics focused on her attractive body line. The same as the above two cases, criticisms of this case centered on two problems; the character looks sexual, and this poster was posted up in public spaces (Ariyoshi, 2019). One critic (Kiiko Ota) claimed that this poster was environmental sexual harassment (OTA, 2019). Also, some critics called for a blood donation boycott (Ariyoshi, 2019). Later, the author of “Uzaki-chan Wants to Hang Out!” expressed that he was a victim of the Great Hanshin-Awaji Earthquake and that this was the reason for collaborating with the Japanese Red Cross Society (Amdo, 2020).

### 2.4. Love-Tights Incident

In November 2022, the official Twitter account of Atsugi Co., Ltd. and a collaboration illustrator held a “Love-tights campaign” on Twitter. Atsugi Co., Ltd. is a Japanese underwear maker and also produces tights. This campaign showed many manga-like illustrations of a female character who wore tights (long underwear). After beginning this campaign, negative opinions against these illustrations appeared on Twitter (The Asahi Shimbun, 2020). Critics said that these illustrations regarded tights as sexual items and that a company that produces tights for female customers must not hold such a campaign (The Asahi Shimbun, 2020). In this case, the company's nature became one important factor that caused the incident. From this case, I have observed that a company's or organization's social role is important when they use female virtual characters. This is one kind of usage context.

### 2.5. Overview

To summarize these cases, I have observed two kinds of opinions that critics have.

- Female characters looking overly sexual itself is a problem.
- The use of sexual female characters in public spaces or by public organizations is a problem.

The first reason is based only on the visual design of virtual characters. The second is influenced by the social context in which virtual characters are used. Here, one question arises; which reason is more important for moe-phobia? Or do these two factors influence human perception through the interaction of the two? This is an important problem in designing virtual agents in the real world. If virtual agents are used for entertainment, sexual appearance may be one point of attraction. However, when the context is public service, will the same agents be effective? This problem has not been researched yet.

In fact, the appearance of characters and the context are possibly interdependent; e.g., a school uniform may generate impressions of both sex appeal and educational context. However, many critics have stated that these two problems are independent in actual moe-phobia controversies. Thus, I think that conducting an experiment focusing on these two factors would be useful.

Therefore, I hypothesize the following.

H: Moe-phobia is associated with not only an agent's appearance (clothes) but also the context (scenario).

In other words, I hypothesize that moe-phobia is associated with three factors: appearance, context, and users' gender.

In this article, I conducted an experiment to investigate the effect of these three factors of female virtual agents on users' perceived resistance. As stated above, the three factors were the agents' appearance, the context in which they are used, and the gender of the participants in the experiment. For appearance, I used agents wearing clothes that were sexual (swimsuit) or not sexual. For context, I used a formal context and an entertainment context. With this experiment, I aimed to verify which factor was larger or if all three factors influenced users' perception through the interaction of the three.

## 3. Materials and Methods

I conducted the experiment with three factors and two levels. The independent variables were the female virtual agents' appearance (business suit and swimwear), the scenario (situation and context) in which they were used, and the participants' gender (female and male). The dependent values were the users' perceptions of the agents.

### 3.1. Agent and Scenario

**Figure 2:**
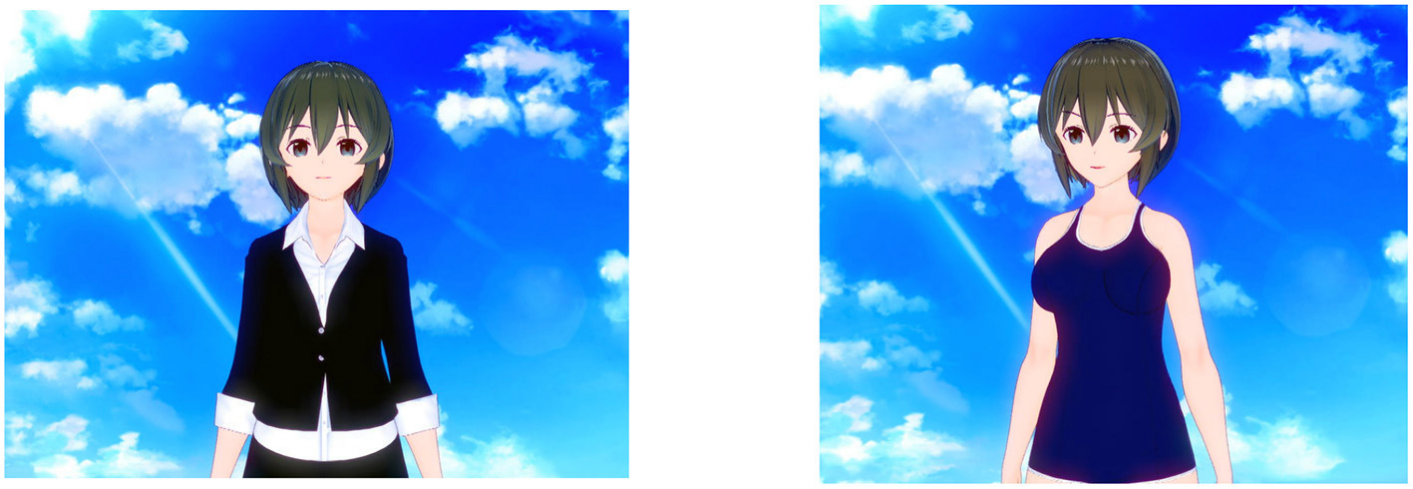
The agent I used in the experiment. The left agent is used for business suit conditions, and the right is for swimsuit conditions. Adapted/Reproduced with permission from IVR, available at https://vkatsu.jp/.

I constructed female virtual agents with V-Katsu, a service for making 3D characters and animation^1^. I constructed two kinds of agents. They had the same facial and physical parts except for their clothes. For the business suit level, the agent wore a dark business suit. For the swimsuit level, the agent wore a swimsuit. This level seemed to be more sexual than the business suit level. These two levels were the appearance factor. Figure 2 shows these two agents.

I used only female agents in this experiment and did not use male agents. This is because, in all actual social cases (refer to Section 2), only female virtual agents were the subject of discussion. So far, moe-phobia has been caused only in regard to female agents. Thus, I focused on only female agents in this article.

Also, I constructed two scenarios in which the agents were used. At the municipal office level, the agents played the role of reception guides in a municipal office. I defined this scenario as a formal context in which the agents were used. For the anime shop level, the agents played the role of a clerk in an anime shop. In both scenarios, the agents spoke different texts. Table 1 shows the text for both levels.

**Table 1:** Speech text for two levels for context.

**Municipal office level**
Hello, everyone. I am working at a reception desk in this municipal office.
This municipal office is open from 9 a.m. to 5 p.m. on weekdays.
You can get a resident's card at the reception on the first floor.
Also, you can get a consultation regarding payment of taxes and social services
on the second floor,
and you can submit for marriage registration and submit a change of
address notification on the third floor.
Also, you can consult with a child development expert every Wednesday.
Please feel free to ask me if there is anything that you don't understand.
**Anime shop level**
Hello, everyone. I am working at a reception desk in this anime shop.
This shop is open from 10 a.m. to 8 p.m.
It's a five-story building, and you can find and get many kinds of anime goods in
this shop.
Also, we often hold a book signing event in the event space on holidays.
Please feel free to ask me if there is anything that you don't understand.

This experimental design was derived on the basis of case studies. In all actual moe-phobia incidents, female agents have been used in public spaces or by public organizations. Also, these agents have traditionally been used in anime or games. Thus, I used a municipal office situation and an anime shop situation for the scenarios.

In both scenarios, the agents were displayed against a blue-sky background as in Figure 2. In this experiment, the agent system, which was designed as if it were appearing on digital signage, and the place (municipal office or anime shop) were assumed to be identifiable from the agents' speech.

Each agent moved as she looked around the audience and moved her lips while speaking.

In the experiment, human-agent interaction was a one-direction interaction (the participants just watched movies). This is because real moe-phobia incidents have mostly been the result of one-direction interactions (TV shows, posters, and SNS advertisements).

I conducted the experiment with four conditions. Table 2 shows two factors for each condition. I created four kinds of movies in which virtual agents spoke for each condition. One participant watched only one movie. After watching the movie, each participant answered questionnaires.

**Table 2:** Levels of two factors for each condition.

	**Agent**	**Scenario (context)**
Condition 1	business suit	municipal office
Condition 2	business suit	anime shop
Condition 3	swimwear	municipal office
Condition 4	swimwear	anime shop

### 3.2. Questionnaires and Analysis

The questionnaires included questions on the agents' trustworthiness, familiarity, likability, sexuality, and suitability as perceived by the participants for each situation. These questions were created by me, and Table 3 shows them. The participants answered these questions on 7-point Likert scales; 1 - strongly disagree, 2 - disagree, 3 - somewhat disagree, 4 - neither agree nor disagree, 5 - somewhat agree, 6 - agree, and 7 - strongly agree.

**Table 3:** Questions I used in the experiment.

**trustworthiness perceived**
Q1: How much did you feel that this agent was trustworthy?
**familiarity perceived**
Q2: How much did you feel that this agent was familiar?
**likability perceived**
Q3: How much did you feel that this agent was likable?
**sexuality perceived**
Q4: How much did you feel that this agent was too sexual?
**suitability perceived**
Q5: How much did you feel that this agent was suitable for this situation
(municipal office/anime shop)?

I conducted a three-way ANOVA for each question in Table 3. The independent values are appearance, context, and participants' gender. I aimed to verify the main effect and interaction of the independent values.

If there was a significant main effect, I conducted a sub-test with the Ryan method. If there was a statistically significant interaction between two factors, I conducted a simple main effect test for the sub-test. Also, if there was a statistically significant interaction between three factors, I conducted a simple main effect test for the sub-test.

### 3.3. Participants

I conducted all experiments on the web. All participants were recruited *via* Yahoo! Crowdsourcing^2^ and received ¥ 50 (about $ 0.46) as a reward. The validity of using crowdsourcing for academic research was shown by Behrend et al. (2011).

For condition 1, I recruited 26 women [ranging in age from 27 to 63 years for an average of 41.8 (SD = 11.3)] and 79 men [ranging in age from 22 to 74 years for an average of 45.1 (SD = 9.4)].

For condition 2, I recruited 17 women [ranging in age from 24 to 70 years for an average of 46.4 (SD = 11.7)] and 48 men [ranging in age from 29 to 86 years for an average of 49.6 (SD = 11.3)].

For condition 3, I recruited 19 women [ranging in age from 20 to 56 years for an average of 37.3 (SD = 10.3)] and 78 men [ranging in age from 26 to 74 years for an average of 47.3 (SD = 10.1)].

For condition 4, I recruited 35 women [ranging in age from 25 to 65 years for an average of 41.7 (SD = 9.91)] and 71 men [ranging in age from 27 to 70 years for an average of 47.5 (SD = 8.8)]. The entire experiment was conducted in accordance with the Ethics Committee of Seikei University and Japanese law. Seikei University granted ethical approval to carry out the study within its facilities (Ethical Application Ref: SREC10-5). All participants were instructed before the experiment that this experiment included virtual characters in sexual clothing.

Also, they were given an explanation about the aim of the experiment.

## 4. Results and Discussion

In all tables, the effect size and statistical power (*p* = 0.05) were calculated *post-hoc*. ** means *p* < 0.01, and * means *p* < 0.05. In all figures, error bars mean standard errors.

**Figure 3:**
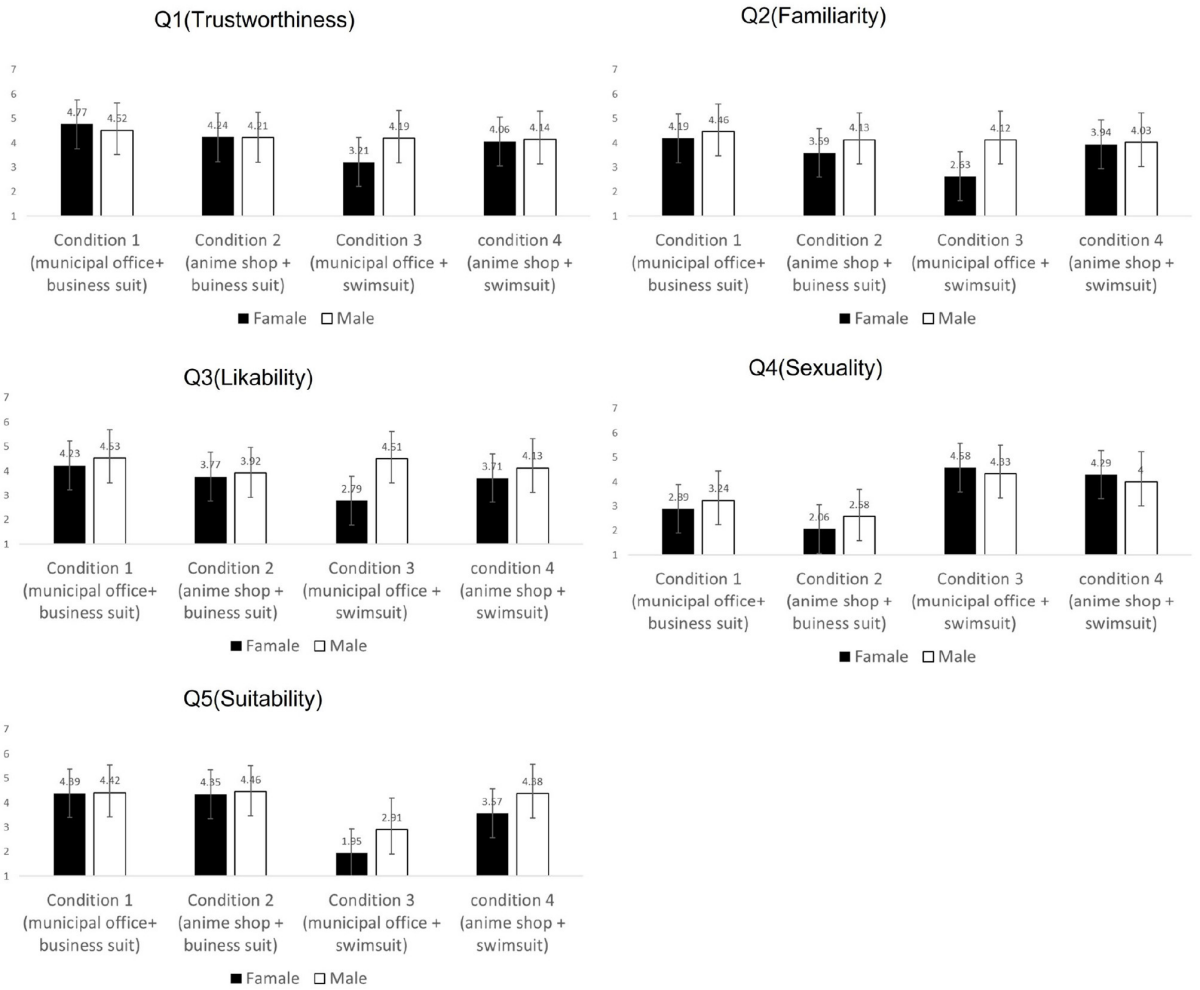
Graphs showing averages for each question observed in the experiment.

Figure 3 shows the averages and SDs for each gender group for Q1-Q5. I conducted a three-way ANOVA for each result. I will explain these results in order.

### 4.1. Trustworthiness

Table 4 shows the results of the three-way ANOVA for Q1 (trustworthiness). There were significant differences in the main effect of appearance (*p* < 0.01). This result shows that the agents in the swimsuit condition were perceived as being less trustworthy than the agents in the business suit condition regardless of the context and the participants' gender.

**Table 4:** Results of three-way ANOVA for Q1 (trustworthiness).

**Source**	***F***	***p***		**Effect size**	**Statistical power**	
**Appearance**	**7.22**	**0.01**	**	**0.140**	**0.773**
Context	0.34	0.56		0.030	0.090
Participants' gender	1.21	0.27		0.058	0.199
Appearance × context	3.41	0.06		0.096	0.462
Appearance × participants' gender	2.99	0.08		0.090	0.415
Context × participants' gender	0.69	0.41		0.043	0.133
Appearance × context × participants' gender	2.71	0.10		0.09	0.386

** means p < 0.01 and bold indicates p < 0.01.

### 4.2. Familiarity

The top of Table 5 shows the results of the three-way ANOVA for Q2 (familiarity). There were statistically significant main effects for the participants' gender (*p* < 0.01) and statistically significant interactions between appearance × context (*p* < 0.05) and appearance × context × participants' gender (*p* < 0.05).

**Table 5:** Results of three-way ANOVA, simple main effect test for appearance × context, and simple main effect test for appearance × context × participants' gender for Q2 (familiarity).

**Source**	***F***	***p***		**Effect size**	**Statistical power**
Appearance	2.89	0.09		0.089	0.403
Context	0.00	0.97		0.002	0.050
**Participants' gender**	**10.24**	**0.00**	**	**0.167**	**0.897**
**Appearance** × **context**	**5.85**	**0.02**	*	**0.126**	**0.684**
Appearance × participants' gender	0.40	0.53		0.033	0.098
Context × participants' gender	1.05	0.31		0.053	0.179
**Appearance** × **context** × **participants' gender**	**6.13**	**0.01**	*	**0.129**	**0.704**
Simple main effect test for appearance × context
**Effect**	***F***	***p***			
**Appearance (context is municipal office)**	**8.49**	**0.00**	**		
Appearance (context is anime shop)	0.26	0.61			
Context (appearance is business suit)	3.02	0.08			
Context (appearance is swimsuit)	2.83	0.09			
Simple main effect test for					
appearance × context × participants' gender					
**Effect**	**F**	***p***			
**Appearance** × **context**
**(participants' gender is female)**	**11.98**	**0.00**	**		
Appearance × context
(participants' gender is male)	0.00	0.97			
**Appearance** × **participants' gender**
**(context is municipal office)**	**4.84**	**0.03**	*		
Appearance × participants' gender
(context is anime shop)	1.70	0.19			
Context × participants' gender
(appearance is business suit)	1.05	0.31			
**Context** × **participants' gender**
**(appearance is swimsuit)**	**6.13**	**0.01**	*		

* means p < 0.05 and ** means p < 0.01. Bold indicates p < 0.05.

**Figure 4:**
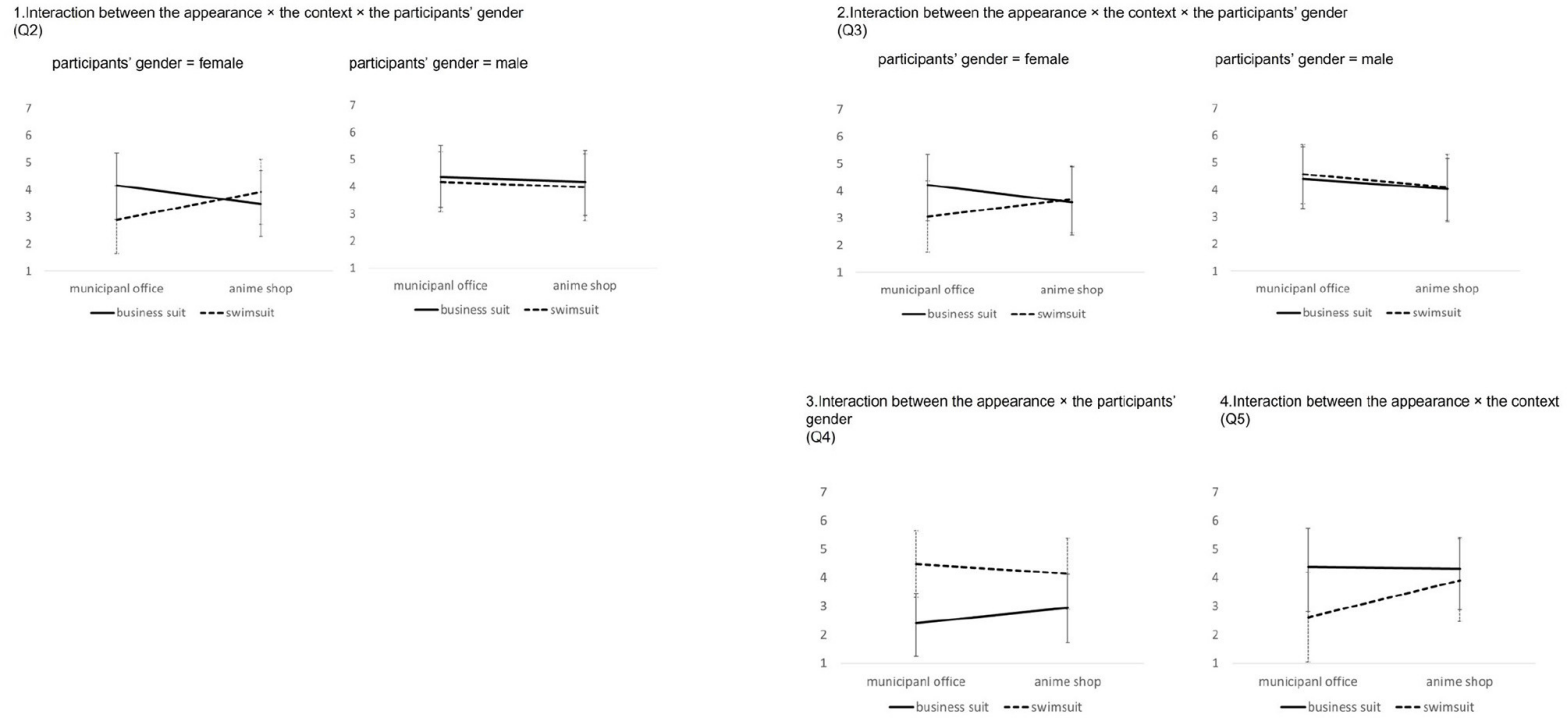
Graphs showing interaction observed in the experiment.

The middle of Table 5 shows the results of a simple main effect test for the interaction between appearance × context. There were significant simple main effects for the appearance when the context was the municipal office (*p* < 0.01). Graph 1 in Figure 4 shows this interaction.

The bottom of Table 5 shows the results of a simple interaction test for the interaction between appearance × context × participants' gender. There were significant differences in the simple interaction between appearance × context when the participants' gender was female (*p* < 0.01), participants' gender × context when the appearance was swimsuit (*p* <0.05), and appearance × participants' gender when the context was the municipal office (*p* < 0.05) Graph 2 in Figure 4 shows this interaction.

Table 6 shows the results of a simple main effect test for each simple interaction. There were significant simple main effects for the appearance when the context was the municipal office and the participants' gender was female (*p* < 0.01). Also, there were significant simple main effects for context when the appearance was a swimsuit, and the participants' gender was female (*p* < 0.01).

**Table 6:** Simple main effect test for appearance × context × participants' gender for Q2 (familiarity).

**Effect**	***F***	***p***			
**Appearance (context is municipal office, participants' gender is female)**	**13.06**	**0.00**	**		
Appearance (context is municipal office, participants' gender is male)	0.26	0.61			
Appearance (context is anime shop, participants' gender is female)	1.64	0.20			
Appearance (context is anime shop, participants' gender is male)	0.31	0.57			
Context (appearance is business suit, participants' gender is female)	3.82	0.051			
Context (appearance is business suit, participants' gender is male)	0.26	0.62			
**Context (appearance is swimsuit, participants' gender is female)**	**8.64**	**0.00**	**		
Context (appearance is swimsuit, participants' gender is male)	0.32	0.57			
Participants' gender (appearance is business suit, context is municipal office)	0.31	0.58			
**Participants' gender (appearance is swimsuit, context is municipal office)**	**13.45**	**0.00**	**		
**Participants' gender (appearance is business suit, context is anime shop)**	**4.03**	**0.05**	*		
Participants' gender (appearance is swimsuit, context is anime shop)	0.03	0.86			

* means p < 0.05 and ** means p < 0.01. Bold indicates p < 0.05.

Also, there were significant simple main effects for the participants' gender when the appearance was a business suit, and the context was an anime shop (*p* < 0.05).

Also, there were significant simple main effect, for the participants' gender when the appearance was a swimsuit, and the context was the municipal office (*p* < 0.01).

These results show that female participants felt more familiarity with the agents in a business suit than the agents in a swimsuit when the agents were used in a municipal office. This tendency was not observed for male participants and the anime shop context.

### 4.3. Likability

The top of Table 7 shows the results of the three-way ANOVA for Q3 (likability). There were statistically significant main effects for the participants' gender (*p* < 0.01) and a statistically significant interaction between appearance × context × participants' gender (*p* < 0.05). The bottom of Table 7 shows the results of a simple interaction test for the interaction of appearance × context × participants' gender. There were statistically significant simple interactions between appearance × context when the participants' gender was female (*p* < 0.01), participants' gender × context when the appearance was swimsuit (*p* < 0.05), and appearance × participants' gender when the context was the municipal office (*p* < 0.01). Table 8 shows the results for a simple main effect test for each simple interaction, and Graph 3 in Figure 4 shows this interaction.

**Table 7:** Results of three-way ANOVA and simple main effect test for appearance × context × participants' gender for Q3 (likability).

**Source**	***F***	***p***		**Effect size**	**Statistical power**
Appearance	1.50	0.22		0.064	0.235
Context	1.56	0.21		0.065	0.242
**Participants' gender**	**13.52**	**0.00**	**	**0.192**	**0.960**
Appearance × context	2.88	0.09		0.08	0.401
Appearance × participants' gender	3.66	0.06		0.100	0.488
Context × participants' gender	1.55	0.21		0.065	0.241
**Appearance** × **context** × **participants' gender**	**4.05**	**0.05**	*	**0.105**	**0.527**
Simple main effect test for
appearance × context × participants' gender
**Effect**	***F***	***p***			
**Appearance** × **context**
**(participants' gender is female)**	**6.88**	**0.01**	**		
Appearance × context
(participants' gender is male)	0.05	0.82			
**Appearance** × **participants' gender**
**(context is municipal office)**	**7.71**	**0.01**	**		
Appearance × participants' gender
(context is anime shop)	0.01	0.94			
Context × participants' gender
(appearance is business suit)	0.30	0.59			
**Context** × **participants' gender**
**(appearance is swimsuit)**	**5.30**	**0.02**	*		

* means p < 0.05 and ** means p < 0.01. Bold indicates p < 0.05.

**Table 8:** Simple main effect test for appearance × context × participants' gender for Q3 (likability).

**Effect**	***F***	***p***			
**Appearance (context is municipal office, participants' gender is female)**	**11.72**	**0.00**	**		
Appearance (context is municipal office, participants' gender is male)	0.08	0.78			
Appearance (context is anime shop, participants' gender is female)	0.25	0.62			
Appearance (context is anime shop, participants' gender is male)	0.04	0.85			
Context (appearance is business suit, participants' gender is female)	3.44	0.06			
Context (appearance is business suit, participants' gender is male)	3.44	0.06			
Context (appearance is swimsuit, participants' gender is female)	1.18	0.28			
Context (appearance is swimsuit, participants' gender is male)	1.97	0.16			
Participants' gender (appearance is business suit, context is municipal office)	0.25	0.62			
Participants' gender (appearance is swimsuit, context is municipal office)	1.60	0.21			
**Participants' gender (appearance is business suit, context is anime shop)**	**19.57**	**0.00**	**		
Participants' gender (appearance is swimsuit, context is anime shop)	1.36	0.24			

** means p < 0.01 and bold indicates p < 0.01.

There were significant simple main effects for the appearance when the context was the municipal office, and the participants' gender was female (*p* < 0.01). Also, there was a significant simple main effect for the participants' gender when the appearance was a swimsuit, and the context was the municipal office (*p* < 0.01).

These results show that the agent in a business suit in a municipal office was more liked by the female participants than the agent in a swimsuit in a municipal office. This suggests that the female users disliked the agent who appeared sexual (swimsuit) in a municipal office. However, this tendency was not observed in the anime shop context. This contradicts the opinion that “female users dislike sexual female agents in all cases.”

### 4.4. Sexuality

The top of Table 9 shows the results of the three-way ANOVA for Q4 (sexuality). There were statistically significant main effects for the appearance (*p* < 0.01) and the context (*p* < 0.01) and a statistically significant interaction between appearance × participants' gender (*p* < 0.05). The significant main effect of the context shows that female and male participants felt the agents used in the municipal office were more sexual than the agents used in the anime shop regardless of the agents' appearance. This possibly refutes the opinion that agents' sexuality is judged on the basis of only appearance.

**Table 9:** Results of three-way ANOVA simple main effect test for appearance × participants' gender for Q4 (sexuality).

**Source**	***F***	***p***		**Effect size**	**Statistical power**
**Appearance**	**80.85**	**0.00**	**	**0.470**	**1.000**
**Context**	**9.33**	**0.00**	**	**0.160**	**0.867**
Participants' gender	0.31	0.56		0.029	0.087
Appearance × context	1.92	0.17		0.072	0.287
**Appearance** × **participants' gender**	**5.98**	**0.01**	*	**0.128**	**0.693**
Context × participants' gender	1.03	0.31		0.053	0.176
Appearance × context × participants' gender	0.10	0.75		0.017	0.062
Simple main effect test
for appearance × participants' gender
**Effect**	***F***	***p***			
**Appearance (participants' gender is female)**	**65.40**	**0.00**	**		
**Appearance (participants' gender is male)**	**21.43**	**0.00**	**		
**Participants' gender (appearance is business suit)**	**4.52**	**0.03**	*		
Participants' gender (appearance is swimsuit)	1.78	0.18			

* means p < 0.05 and ** means p < 0.01. Bold indicates p < 0.05.

There were statistically significant interactions between the appearance × participants' gender. The bottom of Table 9 shows the results for a simple-main effect test. Graph 4 in Figure 4 shows this interaction. There were significant simple main effects for the appearance when the participants' gender was female (*p* < 0.01) and male (*p* < 0.01). Also, there were significant simple main effects for the participants' gender when the appearance was business suit (*p* < 0.05). These results show that both the female and male participants felt that the agent in a swimsuit was more sexual. This may be due to the agents' age and body shape. In any case, this result shows a gender difference in the agents' perceived sexuality. Additionally, Figure 3 shows that the averages for perceived sexuality were higher when the context was a municipal office than when the context was an anime shop for the male participants. This result is counterintuitive, and few prior studies have found similar results. This suggests that “agents' sexuality perceived” was affected by not only the appearance but also the context, and agents in a formal context were actually perceived as being more sexual by male users.

### 4.5. Suitability

The top of Table 10 shows the results of a three-way ANOVA for Q5 (suitability). There were statistically significant main effects for the appearance (*p* < 0.01), the context (*p* < 0.01), and the participants' gender (*p* < 0.05). Also, there were statistically significant interactions between appearance × context (*p* < 0.01). The significant main effects for the participants' gender show that the male participants felt the agents be more suitable than the female participants did regardless of their appearance and the context. This is because there were significant interactions between appearance × context. The bottom of Table 10 shows the results of a simple main effect test. There were significant simple main effects for the appearance when the context was the municipal office (*p* < 0.01). This shows that the participants felt the agent in a business suit to be more suitable than the agent in a swimsuit when the context was the municipal office. Also, there were significant simple main effects for the context when the appearance was swimsuit (*p* < 0.01). This shows that the participants felt the agent in a swimsuit to be more suitable in an anime shop than in a municipal office. Graph 5 in Figure 4 shows this interaction.

**Table 10:** Results of three-way ANOVA simple main effect test for appearance × context for Q5 (suitability).

**Source**	***F***	***p***		**Effect size**	**Statistical power**
**Appearance**	**36.06**	**0.00**	**	**0.314**	**0.999**
**Context**	**11.37**	**0.00**	**	**0.176**	**0.925**
**Participants' gender**	**6.26**	**0.01**	*	**0.131**	**0.713**
**Appearance** × **context**	**14.31**	**0.00**	*	**0.198**	**0.968**
Appearance × participants' gender	2.96	0.09		0.090	0.411
Context × participants' gender	0.050	0.82		0.012	0.056
Appearance × context × participants' gender	0.19	0.66		0.023	0.073
Simple main effect test
for appearance × context
**Effect**	***F***	***p***			
**Appearance (context is municipal office)**	**47.90**	**0.00**	**		
Appearance (context is anime shop)	2.47	0.12			
Context (appearance is business suit)	0.09	0.77			
**Context (appearance is swimsuit)**	**25.59**	**0.00**	**		

* means p < 0.05 and ** means p < 0.01. Bold indicates p < 0.05.

### 4.6. Age Effect

Finally, I investigated whether the age of the participants affected the results or not. I calculated the correlation coefficients between the age of the participants and each of their answers to each question for each condition. The results are shown in Table 11. All correlation coefficients were less than 0.3; thus, I concluded that the effect of age was very small in this experiment. The prior studies showed that users felt empathy more strongly toward virtual agents who appear to be the same age as themselves than other agents (Hosseinpanah et al., 2018). These studies showed that the age of the virtual agents affected users' perception; however, no such effect was observed in my experiment. This suggests that the agents' age did not affect their perceived sexuality and suitability.

**Table 11:** Correlation coefficients between the age of participants and each of their answers.

**Female**		
**Condition**	**Q**	**R**
Condition 1		
	Q1	0.173
	Q2	0.223
	Q3	0.070
	Q4	–0.060
	Q5	0.162
Condition 2		
	Q1	0.132
	Q2	0.241
	Q3	0.182
	Q4	0.235
	Q5	0.182
Condition 3		
	Q1	0.131
	Q2	0.168
	Q3	0.154
	Q4	–0.121
	Q5	0.208
Condition 4		
	Q1	0.157
	Q2	0.128
	Q3	0.164
	Q4	–0.108
	Q5	0.190

### 4.7. Overview

In summary, for agents' familiarity, likability, and suitability, I observed a simple main effect for the participants' gender.

In particular, for the suitability, both female and male participants tended to feel that the agent in the business suit was more suitable for the municipal office than the agent in swimwear and that the agent in swimwear was more suitable in the anime shop than in the municipal office. This is the most important result of this experiment. The female and male participants tended to have different impressions of the agents; however, they tended to have the same perception of the agents' suitability. This result suggests that the agents in sexual clothes (swimwear) and a formal context (municipal office) were not accepted by the female and male participants. Also, the swimwear and the anime shop itself did not negatively affect the agents' perceived suitability; only the combination of the two generated a negative effect. From prior studies, only appearance or sexual visual symbols were indicated as the reason for there being an impression of sexuality (Huo and Yuan, 2017; Wirtz et al., 2018). Küster et al. showed that participants judged female virtual agents by what they wore (Küster et al., 2019). Their experiment was conducted without any verbal information. My findings also show that what they wear is important; however, this effect was influenced by context and the participants' gender. My finding redefined this model.

Second, this result suggests an important aspect in designing virtual agents to avoid moe-phobia. Sexual clothes do not always lead to moe-phobia; the context (situation) is another important factor for moe-phobia. Thus, the context in which these agents are used must also be considered. Also, the results of this experiment show that an actual controversy may be caused by not only the agents' appearance but also the situation in which the agents are used. In this experiment, the female and male participants did not judge the suitability of the agents from only the agents' clothes. Casual situations may allow the use of an agent in swimwear, while formal situations may not. Also, in advertisements, sexual visual symbols are widely used and can have a positive effect (Huo and Yuan, 2017; Wirtz et al., 2018). The findings show that a sexual female virtual agent will have a negative effect in some contexts. In summary, moe-phobia was substantially caused by the context.

Third, this result seems to be able to explain the contradiction among previous studies regarding the gender of agents and participants. Previous studies that focused on agents' and participants' gender had contradictory results (Guadagno et al., 2007; Kim et al., 2007; Payne et al., 2013). This finding suggests that these different results may be caused by the context in which the agents were used. Also, I suggest that a study on the impression given by agents' gender (especially sexuality and suitability) should consider the experimental context.

Finally, this finding suggests a way of avoiding moe-phobia incidents; social context is as important as the agents' appearance. To avoid moe-phobia, we should design virtual characters considering the context and users' gender.

### 4.8. Limitations

This article has some limitations. The agents and scenarios were limited in the experiment. In particular, I used only female agents and did not consider male ones. If I were to add male agents and look at all gender interactions (female and male participants, female and male agents), I would possibly look at the relationship between agents' sexuality and participants' perception of it. Also, it is an important problem to determine whether these results are particular to Japanese participants or not. To solve this problem, I have to conduct a cross-cultural experiment. Also, moe-phobia in women may have other causes, e.g., opposition to gender stereotypes. For example, the Love-tights incident seemed to be partly caused by opposition to the idea that tights symbolize female sexuality.

## 5. Conclusion

In this article, I focused on incidents involving virtual agents that were associated with the sexuality of agents as perceived by people. I referred to this phenomenon as “moe-phobia.” After discussing actual incidents, I hypothesized that moe-phobia is associated with not only the appearance of agents but also the situations in which they are used. Thus, I planned an experiment with three factors and two levels. The independent values were the agents' clothes (business suit and swimwear) and their usage scenario (municipal office and anime shop). I created four kinds of movies in which virtual agents spoke for each condition. Also, I conducted the experiment with a group of female participants and a group of male participants. In the experiment, the participants watched one movie and answered questions about the agents' trustworthiness, familiarity, likability, sexuality, and suitability perceived. I conducted a three-way ANOVA on each score for the female and male groups. As a result, I observed a different tendency in the impression of the agents between the female and male groups; however, both groups had the same tendency regarding suitability. The female and male participants judged the agents' suitability from not only the agents' clothes but also the scenario. This result suggests new guidelines for designing virtual agents that are used in public spaces.

## Data Availability Statement

The original contributions presented in the study are included in the article/Supplementary Material, further inquiries can be directed to the corresponding author.

## Ethics Statement

The studies involving human participants were reviewed and approved by Seikei University. Written informed consent for participation was not required for this study in accordance with the national legislation and the institutional requirements.

## Author Contributions

TM came up with the model and experimental design, conducted the experiments and analysis, drafted the manuscript, and participated in the review and revision of the manuscript and has approved the final manuscript for publication.

## Funding

This research was partially supported by JSPS KAKENHI (no. 20H05571).

## Conflict of Interest

The author declares that the research was conducted in the absence of any commercial or financial relationships that could be construed as a potential conflict of interest.

## Publisher's Note

All claims expressed in this article are solely those of the authors and do not necessarily represent those of their affiliated organizations, or those of the publisher, the editors and the reviewers. Any product that may be evaluated in this article, or claim that may be made by its manufacturer, is not guaranteed or endorsed by the publisher.

## Footnotes

^1^ https://vkatsu.jp/

^2^ https://crowdsourcing.yahoo.co.jp/
